# A 2.9 Mb Chromosomal Segment Deletion Is Responsible for Early Ripening and Deep Red Fruit in *Citrus sinensis*

**DOI:** 10.3390/ijms252312931

**Published:** 2024-12-02

**Authors:** Jianmei Chen, Zhenmin Chen, Quming Xie, Xiaotong Wu, Qingyu Pei, Yi Lin, Qiong Chen, Shubei Wan

**Affiliations:** National Navel Orange Engineering Research Center, College of Life Sciences, Gannan Normal University, Ganzhou 341000, China; chenjm@gnnu.edu.cn (J.C.); chen002529@163.com (Z.C.); xqming78@163.com (Q.X.); wxt180860@163.com (X.W.); peiqingyu392766@163.com (Q.P.); 1221016007@gnnu.edu.cn (Y.L.); qiong0552@163.com (Q.C.)

**Keywords:** *Citrus sinensis*, structural variation, fruit ripening, fruit color

## Abstract

Sweet orange (*Citrus sinensis*) is an economically important fruit crop worldwide. Mining for genes associated with ripening periods and fruit color traits is crucial for plant genetics and for the improvement of external fruit quality traits. The present study identified a novel navel orange accession, designated as Ganhong, with early ripening and deep red fruit traits. DNA sequence analysis showed a 2.9 Mb deletion in one copy of chromosome 7 in Ganhong navel orange. Flesh samples from Ganhong and its parental variety, Newhall navel orange, were sampled for RNA sequence analysis 200 days after flowering; 958 differentially expressed genes (DEGs) were identified between the two varieties. Functional enrichment analysis indicated that these DEGs were mainly enriched in phytohormones, particularly abscisic acid (ABA), related to fruit ripening. The deletion interval has 343 annotated genes, among which 4 genes (*Cs_ont_7g018990*, *Cs_ont_7g019400*, *Cs_ont_7g019650*, and *Cs_ont_7g019820*) were inferred to be candidate causal genes for early ripening and deep red fruit traits based on gene functionality and gene expression analysis. The present study laid a foundation for further elucidation of the mechanisms underlying the early ripening and deep red fruit trait in Ganhong navel orange.

## 1. Introduction

Navel orange is an economically important citrus fruit crop and is widely grown in south Jiangxi Province (Gannan), China. Gannan navel orange shows a single variety structure, and the Newhall variety is estimated to account for 90% of the cultivation area [1,2]. This presents significant challenges with regard to production and market consumption, particularly due to the high concentration of supply during the brief fruit ripening period, which diminishes market competitiveness. Consequently, the selection of high-quality navel orange varieties is essential for enhancing the structural adjustment of the navel orange industry and fostering the economic development of the cultivation regions. Navel orange genetics, genomics of mutants, and mining of important functional genes have generated significant interest in the molecular breeding of new citrus cultivars.

Relatively little is known about the regulatory mechanisms underlying ripening in citrus fruits, which are non-climacteric fruits. Plant hormones play important roles in fruit development and are key regulators of fruit ripening [3]. Ripening of non-climacteric fruits is associated with abscisic acid (ABA) [4]. However, the detailed molecular mechanisms are not yet understood. Recently, several late-/early-ripening citrus mutants have been developed to study the molecular mechanisms of ripening using omics analysis. Research in these mutants was performed to investigate the roles of genes and noncoding RNAs related to various pathways involved in fruit ripening, such as sugar metabolism and plant hormones [5,6,7,8]. However, the molecular regulatory networks of citrus fruit ripening remain unclear and require further intensive research.

Fruit color is an important trait contributing to fruit quality and market value. At the ripening stage, citrus fruits exhibit a deep orange or reddish color depending on the concentrations of carotenoids and flavonoids [9,10]. There have been reports of several molecular mechanisms that contribute to fruit color in citrus. Two transcriptional regulatory modules, *CsERF110-CsERF53* and *CsHB5-CsbZIP44*, were reported to be responsive to ABA signaling, thereby orchestrating citrus fruit coloration by regulating carotenoid metabolism [11,12]. Multiple genes, including *CsTT8* (Testa Transparent 8), *CsMADS3* (MADS-box transcription factor), *CsPHL3* (Pi starvation response factor (PHR)-like), *CsERF110* (ethylene-responsive transcription factor), *CsERF53*, and *CsERF61*, were shown to promote fruit coloration in citrus by regulating carotenoid metabolism [11,13,14,15]. The MYB regulatory gene *Ruby* can facilitate the accumulation of anthocyanins, which causes fruit coloration [16]. These previous studies indicated the involvement of a complex gene regulatory network in the determination of fruit color.

Structural variation (SV) is defined as a change in the structure of a chromosome larger than 50 bp and includes insertions, deletions, inversions, duplications, and interchromosomal translocations [17]. In plants, SV plays an important role in promoting genome evolution and phenotypic changes [18,19]. In peach, a 1.7 Mb chromosomal inversion downstream of the gene encoding PpOFP1 (OVATE Family Protein 1), which interacts with fruit elongation activator PpTRM17 (TONNEAU1 recruiting motif protein), is responsible for flat fruit shape [20]. A total of 2321 somatic SVs were detected in 114 sweet orange mutants, some of which were shown to be associated with fruit acidity; for example, a 6.9 kb insertion upstream of a Na^+^/H^+^ transporter (*CsNHX*) was shown to be associated with low acidity [21]. Transposable element-mediated chromosomal rearrangements, including deletions and inversions, were observed in two mutants derived from *Citrus clementine* [22].

Here, we report a new *Citrus sinensis* line designated as Ganhong with early ripening and deep red fruit. With this novel accession, we identified and characterized a structural deletion involved in citrus fruit ripening and coloration through analysis and comparison of the genomes of Ganhong and the parental variety, Newhall navel orange, and identified the candidate key genes for these traits based on gene expression analyses and functional annotation. Further, we examined the mechanisms underlying the early ripening and coloration of Ganhong navel orange through comparative transcriptomics analysis. Our findings will help to elucidate the mechanisms underlying the early ripening and deep red fruit trait and provide novel insights into the formation mechanism of structural variation in citrus.

## 2. Results

### 2.1. Fruit Ripening Period and Morphology of Ganhong Navel Orange

**Figure 1:**
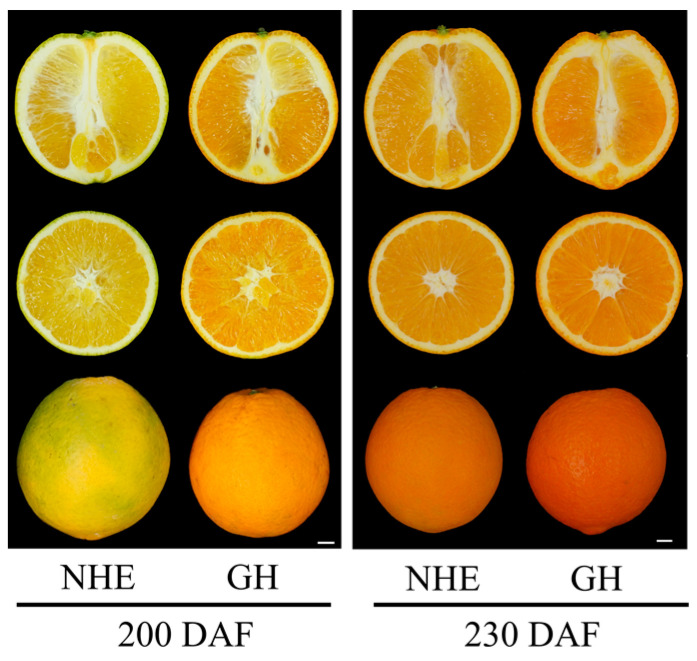
Performance of fruits of Ganhong (GH) and Newhall (NHE) navel orange at 200 and 230 days after flowering (DAF), scale bars = 1 cm.

The Ganhong navel orange originated from a bud mutation of the Newhall navel orange. Ganhong color turned 4 weeks earlier compared with the original variety (Figure 1, Figure S1). The ripening Ganhong fruit peel and flesh were significantly redder than those of Newhall fruit (Figure 1). Colorimetry values of ripening fruit peels of the two varieties were measured and are presented in Table 1. The color parameters L*, a*, and b* represent light to dark, red to green, and yellow to blue color, respectively. The a* values of Ganhong were significantly higher, whereas the L* and b* values of Ganhong were significantly lower than those of Newhall navel orange. These results suggested that Ganhong navel orange is an early-ripening cultivar with redder peel and flesh.

### 2.2. Whole-Genome Resequencing Analysis of Ganhong Navel Orange

**Figure 2:**
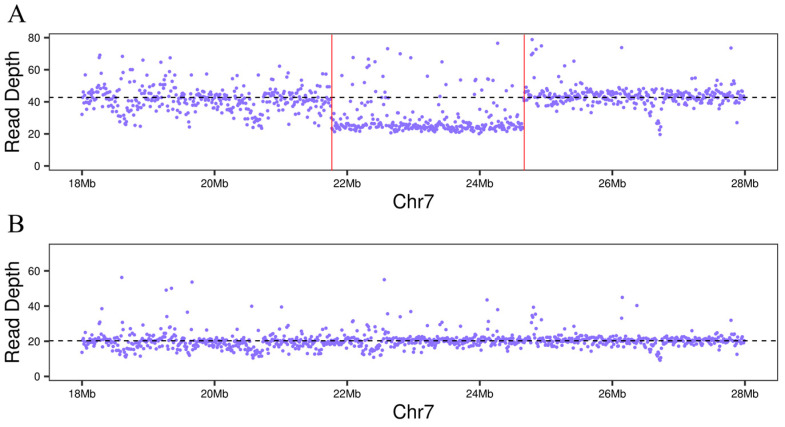
The read depth of Ganhong (**A**) and Newhall (**B**) revealed the large deletion on chromosome 7.

The genomes of Ganhong and Newhall navel orange were resequenced on the HiSeq 3000 platform (Illumina, San Diego, CA, USA). For mapping, the high-quality sequence of the C. sinensis V3.0 reference genome (http://citrus.hzau.edu.cn, accessed on 1 December 2024) was used as a reference. The average depths of genome sequencing of Ganhong and Newhall navel orange were 40× and 20×, respectively. The sequences of Ganhong and Newhall navel orange were uploaded to NCBI with the study accession number PRJNA1168425. Chromosomal rearrangements were investigated through read depth. The coverage profiles for any given chromosome were remarkably similar in the genomes of Ganhong and Newhall navel orange, except in a fragment of chromosome 7 of Ganhong that showed reduced depth (29.6×), suggesting the occurrence of putative gross deletions (Figure 2, Figure S2). Integrative Genomics Viewer (IGV) was used to examine the two breakpoints of the deletion manually. Two candidate breakpoints located at Chr7: 21749629 and Chr7: 24651989 were identified (Figure S3).

### 2.3. Verification of Novel Segment Deletion by PCR

**Figure 3:**
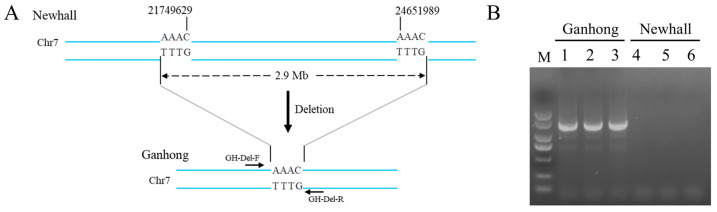
Differences in sequence and structural characteristics of one of Chr7 chromosomes between Ganhong and Newhall navel orange and verification of novel segment deletion by PCR. (**A**) The regular chromosome segment without deletion in Newhall and a mutant chromosomal segment with 2.9 Mb deletion in Ganhong navel orange. (**B**) Experimental results of marker GH-Del scan with Ganhong and Newhall. The 1460 bp bands of GH-Del were detected in Ganhong plants rather than in Newhall plants. M, marker DL2000.

Based on the deletion breakpoint sequences, a pair of primers (GH-Del-F/R) were designed to cover the region of flanking sequences on either side of the deletion for verification of the novel segment deletion in Chr7 (Figure 3A). A band of 1460 bp was detected only in Ganhong plants (Figure 3B). The specific band was confirmed to represent a novel segment deletion in Chr7 in Ganhong navel orange by sequence analysis. Our results strongly suggested that a 2.9 Mb segmental deletion in one copy of Chr7 in Ganhong navel orange was responsible for the early ripening and deep red fruit traits (Figure 3A).

### 2.4. RNA-Seq Analysis

To identify transcriptomic changes resulting from the deletion, we performed RNA-seq analysis of Ganhong and Newhall navel orange flesh samples 200 days after flowering. Differentially expressed genes (DEGs) were filtered using thresholds of |log2(FC)| ≥ 1 and FDR < 0.05. A total of 958 genes showing significant differential expression were identified, including 518 upregulated genes and 440 downregulated genes (Figure S4, Table S1). Of the DEGs, two upregulated genes and thirty-one downregulated genes mapped to the deleted segment in Ganhong navel orange.

**Figure 4:**
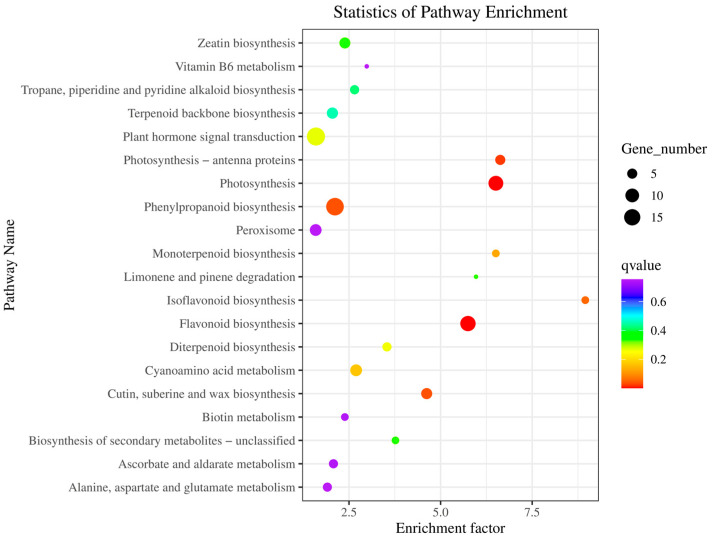
KEGG pathway enrichment analysis for differentially expressed genes.

In the present study, we also analyzed the key pathways for the regulation of early ripening and deep red fruit traits in Ganhong and Newhall navel orange. Kyoto Encyclopedia of Genes and Genomes (KEGG) pathway enrichment analysis was performed on the DEGs. We identified the top 20 KEGG pathway enrichment terms as shown in Figure 4. There were twelve and five DEGs significantly enriched in the ‘Photosynthesis’ and ‘Photosynthesis-antenna proteins’ pathways, respectively. All DEGs associated with these pathways were downregulated and possibly related to the color alteration of the flesh of Ganhong oranges.

Phytohormones, particularly ABA, have been shown to participate in the regulation of citrus fruit repining and coloration [8,23]. A total of 19 DEGs were enriched in the ‘Plant hormone signal transduction’ pathway. Among the DEGs, *Cs_ont_2g002030* [9-cis-epoxycarotenoid dioxygenase (NCED)], encoding a key enzyme for ABA biosynthesis, was significantly upregulated in the flesh of Ganhong fruit. Genes encoding ABA receptors, including *Cs_ont_1g021530* [ABA-activated protein kinase 2 (SAPK2)], *Cs_ont_5g004380* (abscisic acid receptor PYL9), and *Cs_ont_7g010910* (abscisic acid receptor PYL1), were differentially expressed in the flesh of fruits of Ganhong and Newhall. These results suggested that ABA plays an important role in the early-ripening trait of Ganhong oranges.

### 2.5. Candidate Key Genes for Early Ripening and Deep Red Fruit Traits in Ganhong

Annotation was performed in the candidate region according to the C. sinensis v3.0 reference genome (http://citrus.hzau.edu.cn/, accessed on 1 December 2024). A total of 343 genes were localized to the deleted interval (Table S2). We analyzed the expression of these 343 genes at different fruit ripening stages using transcriptomics data from previous studies [8,24]. A detailed list of the 343 genes and their expression levels in flesh and peel during fruit development and ripening is presented in Table S2.

**Figure 5:**
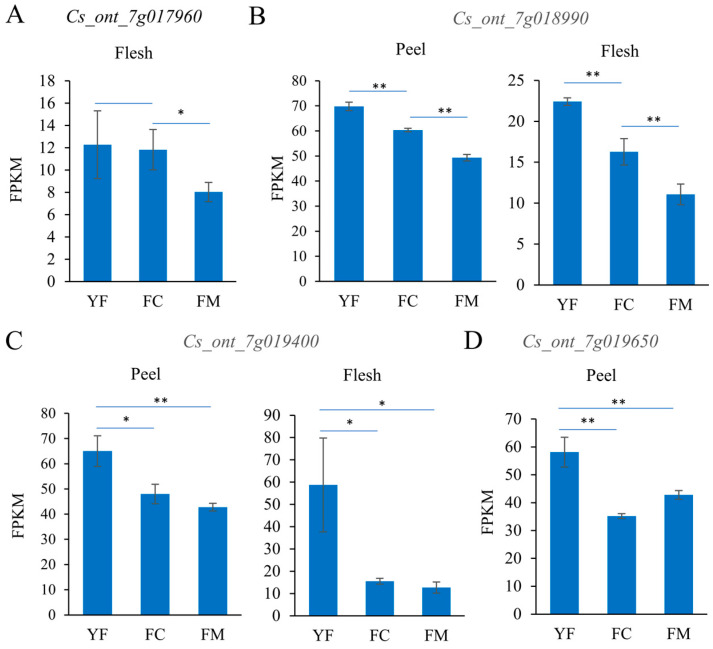
Expression characterization of four genes in fruits at different developmental stages. (**A**) *Cs_ont_7g017960*; (**B**) *Cs_ont_7g018990*; (**C**) *Cs_ont_7g019400*; (**D**) *Cs_ont_7g019650*; ‘YF’, ‘FC’, and ‘FM’ represent young fruit, fruit-coloring onset, and mature fruit, respectively. Values are means ± SDs (*n* = 3). * and ** denote significant differences at the 0.05 and 0.01 probability levels, respectively.

Four genes, *Cs_ont_7g018990*, *Cs_ont_7g019400*, *Cs_ont_7g019650*, and *Cs_ont_7g019820*, were identified as candidate key genes for early ripening and deep red fruit traits in Ganhong oranges by gene expression profiling and functional annotation (Table 2). Compared to Newhall, the expression levels of these four genes showed significant decreases in the flesh of Ganhong at the 200 DAF stage (Table 2). *Cs_ont_7g017960* encoding carotenoid cleavage dioxygenase 4 (CCD4) was proposed to participate in the regulation of β-carotenoid degradation [25]. Compared to the young fruit and fruit-coloring onset stage, the expression level of *Cs_ont_7g017960* in flesh at the mature fruit stage showed significant decreases in C. sinensis (Figure 5A). *Cs_ont_7g018990* encodes TCP (Teosinte branched1/Cycloidea/Proliferating cell factor) transcription factor 7. In strawberry, TCP7 was shown to negatively regulate fruit ripening in response to ABA controlling sugar accumulation [26]. As the fruit developed and ripened, the expression level of *Cs_ont_7g018990* decreased gradually in the flesh and peel of C. sinensis (Figure 5B). *Cs_ont_7g019400* encodes ERF119 (ethylene-responsive transcription factor119). Compared to the young fruit stage, the expression level of *Cs_ont_7g019400* at the onset of fruit coloring and the mature fruit stage showed significant decreases in the flesh and peel of C. sinensis (Figure 5C). *Cs_ont_7g019650* encodes a PIN-LIKES 3 protein, which was reported to be a member of the putative auxin transport facilitator family and could regulate the balance of auxin [27]. Compared with the young fruit stage, the level of *Cs_ont_7g019650* expression in flesh at the onset of fruit coloring and the mature fruit stage showed significant decreases in C. sinensis (Figure 5D). Other genes in the deleted chromosomal segment could not be completely excluded from the list of candidate genes, although they have not been associated with fruit ripening and coloration.

## 3. Discussion

To better meet the growing market demand, the citrus industry has been attempting to develop new cultivars with specific characteristics, such as early ripening and new colors [8,28,29,30]. In this study, Ganhong navel orange was identified as a novel cultivar of C. sinensis. This new cultivar has stable early ripening and deep red fruit and is considered a suitable candidate for citrus fruit under current and future markets.

Chromosomal structural variations have been reported to contribute to variations in complex traits by affecting gene expression [21,31]. There have been few reports of trait variation resulting from the deletion of large chromosome segments in plants, with research primarily concentrating on chromosome inversions or inserts/deletions of smaller segments. Such phenomena may occur due to the deletion of substantial chromosomal regions, leading to alterations in the expression levels of multiple genes and their subsequent elimination during the course of evolution. However, large deletions have been found in some Rutaceae citrus species, including sweet orange and clementine [21,22]. Here, we reported a novel large chromosomal segment deletion mutant in C. sinensis. The preservation of this large deletion in Ganhong during selection was likely due to asexual propagation and heterozygosity of the deletion.

Hybridization and meiosis are important means of generating chromosomal structural variations [32,33,34]. Navel orange is an asexually propagated crop that cannot be outcrossed. Ganhong navel orange was originally isolated as a bud mutation from the Newhall navel orange. The 2.9 Mb deletion in one of the Chr7 pairs in Ganhong navel orange may have occurred during mitosis.

The most important mechanisms of chromosomal rearrangements include non-allelic homologous recombination (NAHR), non-homologous end joining (NHEJ), and fork stalling and template switching/microhomology-mediated break-induced replication (FoSTeS/MMBIR) [18,35,36]. NAHR generally occurs at large inverted repeats, and it is widely accepted that transposable element-mediated NAHR requires coordinated transposition or involvement of at least two transposable elements [18,22,31]. Unlike NAHR, the NHEJ pathways repair (double-strand breaks) DSBs by directly ligating the two broken DNA ends. One kind of NHEJ is microhomology-mediated end joining (MMEJ), which utilizes shorter regions of imperfect or interrupted microhomologies at the breakpoints of microhomology [35,36]. FoSTeS/MMBIR is the result of mechanisms for repairing DNA replication errors and caused the deletion during two microhomologies. In this study, sequence alignment revealed no inserted homologous transposable element sequences at two deletion breakpoints, but four base pairs of a microhomology sequence (AAAC) were found at both breakpoints in Ganhong (Figure 3A). The DNA replication FoSTeS/MMBIR mechanism seemed to better explain the formation of this novel deletion mutation.

## 4. Materials and Methods

### 4.1. Plant Materials

Ganhong navel orange was a new early-ripening variety and was originally isolated as a bud mutation from the Newhall navel orange in Ganzhou, China. All plant materials were grown in the fields of the experimental nursery at Gannan Normal University and planted under the same conditions. The plants were grown and managed in accordance with regular agricultural practices. Pulp samples for RNA-seq analysis were collected from Ganhong and Newhall plants at 200 DAF. Three independent biological replicates were performed. Samples were immediately frozen in liquid nitrogen and stored at −80 °C until use.

### 4.2. Quantification of Fruit Peel Colorimeter

The mature fruit color differences of the two varieties were measured by an NR200 colorimeter (3NH, Shenzhen, China). Accordingly, the color of composites was expressed in terms of 3 coordinate values (L*, a*, b*), where L* represents brightness ranging from 0 to 100, ‘a*’ represents red (0~+128) or green chromaticity (−128~0), and ‘b*’ is the color of yellow (0~+128) or blue chromaticity (−128~0).

### 4.3. DNA Extracted and Whole-Genome Re-Sequencing

Genomic DNA was extracted from young leaves of Ganhong and Newhall navel orange by the CTAB method [37]. Whole-genome resequencing of two germplasms was carried out on a HiSeq 3000 platform (Illumina, San Diego, CA, USA). Raw data were preprocessed using the fastp (0.20.0) tool (http://github.com/openGene/fastp, accessed on 1 December 2024). These clean reads were mapped to the reference genome (*Citrus sinensis* v3.0) using BWA (v0.7.17) software [38]. Genome Analysis Toolkit (GATK) software (https://software.broadinstitute.org/gatk/; 4.1.4.1, accessed on 1 December 2024) was used to identify genetic variants [39]. Two deletion breakpoints were individually visualized on Integrative Genomics Viewer (IGV) software (http://www.broadinstitute.org/igv/, accessed on 1 December 2024).

### 4.4. Markers Based on Novel Segment Deletion

A pair of GH-Del primers were designed based on the following deletion breakpoints: GH-Del-F (5′-GTGCCGCAACTGACCATG-3′) and GH-Del-R (5′-AGGATTTGTAATCCATCAAATAAGG-3′). PCR was performed in a 25 μL reaction volume using 2 × Phanta Flash Master Mix (Dye Plus) (Vazyme, Nanjing, China) according to instructions. Then, the polymorphic bands were separated on an agarose gel (1%). Specific bands were recovered using a gel recovery kit (Vazyme, Nanjing, China) and sent to Tsingke Biotechnology Co., Ltd. (Nanjing, China) for DNA sequencing.

### 4.5. RNA Preparation

Total RNA was extracted from pulp tissues using the FastPure Universal Plant Total RNA Isolation Kit (Vazyme, Nanjing, China). RNA integrity and concentration were verified by an Agilent 2100 bioanalyzer (Agilent, Santa Clara, CA, USA) and spectrophotometry (Nanodrop ND-2000, Thermo Fisher Scientific, Waltham, MA, USA). After extraction, RNA was stored at −80 °C.

### 4.6. RNA-Seq Analysis

Whole-transcriptome (RNA-Seq) analysis of 200 DAF Ganhong pulp tissue was performed using 200 DAF Newhall pulp tissue as the control. Library construction was conducted using the library building kit (Illumina, San Diego, CA, USA). RNA-Seq and computational analysis were performed using the Illumina NovaSeq platform. The experiment was conducted with three biological replicates. Clean reads were mapped to the reference genome (*Citrus. sinensis* v3.0; http://citrus.hzau.edu.cn, accessed on 1 December 2024) using HISAT2 (v2.1.0) software [40]. The differential expression gene was analyzed using DESeq2 (v1.26.0) [41]. The genes with an adjusted |log_2_(FC)| ≥ 1 and FDR < 0.05 were regarded as DEGs. Functional enrichment analysis of DEGs was performed by mapping each DEG to GO and KEGG databases [42,43]. GO enrichment analysis was performed using the Blast2GO (v.5.2.5) tool [43].

### 4.7. Expression Analysis of the Genes Which Were Located on the Deleted Interval

The RNA-seq data of orange fruit flesh at young fruit, fruit-coloring onset, and the fruit delayed-harvest stage were downloaded from the NCBI sequence read archive (SRA) with accession code PRJNA387319. The transcriptome data of fruit peels at three developmental stages (180, 200, and 220 DAF) were obtained from previously published studies [8]. Fragments per kilobase of exon model per million mapped reads (FPKM) were used to measure the transcript abundance of the genes.

## 5. Conclusions

A new navel orange accession with early ripening and deep red fruit, Ganhong, was discovered from our *Citrus sinensis* germplasm. DNA sequencing analysis studies showed that the early ripening and deep red fruit trait was controlled by a 2.9 Mb segmental deletion in one of the two Chr7 chromosomes. Four genes, *Cs_ont_7g018990*, *Cs_ont_7g019400*, *Cs_ont_7g019650,* and *Cs_ont_7g019820*, were identified as candidate key genes for early ripening and deep red fruit based on gene expression and functional analyses. Further analysis of comparative transcriptomics revealed that ABA plays an important role in the formation of early-ripening formation in Ganhong oranges. This study provides a theoretical foundation for elucidating the ripening period, fruit color mechanism, and breeding citrus. Meanwhile, our results provide scientific evidence for the promotion of promising early-ripening navel orange cultivars Ganhong. To conclude, this research is expected to contribute to the development of the citrus industry.

## Figures and Tables

**Table 1:** Color quality attributes of ripening fruit peels of Ganhong (GH) and Newhall (NHE) navel orange.

	L*	a*	b*
Newhall	64.73 ± 1.29	24.95 ± 1.53	45.08 ± 2.12
Ganhong	59.99 ± 1.44 **	30.07 ± 0.86 **	34.79 ± 2.28 **

**Notes**: ** Indicates a highly significant difference (q-value < 0.01) judged by Student’s *t*-test.

**Table 2:** List of candidate key genes for the early ripening and deep red fruit traits in Ganhong.

Gene ID	Homolog in *Arabidopsis thaliana*	Putative Molecular Function	NHE	GH
*Cs_ont_7g017960*	*AT4G19170*	Carotenoid cleavage dioxygenase 4 (CCD4)	8.29 ± 0.61	6.81 ± 0.39 *
*Cs_ont_7g018990*	*AT5G23280*	Transcription factor TCP7	26.71 ± 2.68	18.52 ± 1.88 *
*Cs_ont_7g019400*	*AT1G68550*	Ethylene-responsive factor ERF119	31.62 ± 1.49	14.18 ± 3.10 **
*Cs_ont_7g019650*	*AT1G76520*	Protein PIN-LIKES 3	37.55 ± 5.49	17.72 ± 3.65 *

**Notes**: * and ** denote significant differences at the 0.05 and 0.01 probability levels judged by Student’s *t*-test, respectively.

## Data Availability

Whole-genome resequencing and RNA-seq data of this study have been uploaded to the National Biotechnology Information Center (NCBI) SRA database with the primary accession code PRJNA1168425.

## Supplementary Materials

The following supporting information can be downloaded at https://www.mdpi.com/article/10.3390/ijms252312931/s1.

## References

[1] Zhang J., Yang Z.Q., Liang Y., Zhang L.Y., Ling W., Guo C., Liang G.L., Luo G.T., Ye Q., Zhong B.L. (2018). Effects of Postharvest Time, Heat Treatment, pH and Filtration on the Limonin Content in Newhall Navel Orange (*Citrus sinensis* Osbeck cv. Newhall) Juice. Molecules.

[2] Zhang J., Zhang J.Y., Shan Y.X., Guo C., He L., Zhang L.Y., Ling W., Liang Y., Zhong B.L. (2022). Effect of harvest time on the chemical composition and antioxidant capacity of Gannan navel orange (*Citrus sinensis* L. Osbeck ‘Newhall’) juice. J. Integr. Agr..

[3] Chen C.Q., Chen H.X., Yang W.L., Li J., Tang W.J., Gong R.G. (2022). Transcriptomic and metabolomic analysis of quality changes during sweet cherry fruit development and mining of related genes. Int. J. Mol. Sci..

[4] Symons G.M., Chua Y.J., Ross J.J., Quittenden L.J., Davies N.W., Reid J.B. (2012). Hormonal changes during non-climacteric ripening in strawberry. J. Exp. Bot..

[5] Wu J.X., Zheng S.S., Feng G.Z., Yi H.L. (2016). Comparative analysis of miRNAs and their target transcripts between a spontaneous late-ripening sweet orange mutant and its wild-type using small RNA and degradome sequencing. Front. Plant Sci..

[6] Pan H.L., Lyu S.H., Chen Y.Q., Xu S.R., Ye J.W., Chen G.X., Wu S.H., Li X.T., Chen J.J., Pan D.M. (2021). MicroRNAs and transcripts Associated with an early ripening mutant of Pomelo (*Citrus grandis* Osbeck). Int. J. Mol. Sci..

[7] Mi L.F., Ma D., Lv S.P., Xu S.B., Zhong B.L., Peng T., Liu D.C., Liu Y. (2022). Comparative transcriptome and sRNAome analyses reveal the regulatory mechanisms of fruit ripening in a spontaneous early-ripening navel orange mutant and its wild type. Genes.

[8] Chen J.M., Xie L.H., Lin Y., Zhong B.L., Wan S.B. (2024). Transcriptome and weighted gene co-expression network analyses reveal key genes and pathways involved in early fruit ripening in *Citrus sinensis*. BMC Genom..

[9] Huang D., Wang X., Tang Z.Z., Yuan Y., Xu Y.T., He J.X., Jiang X.L., Peng S.A., Li L., Butelli E. (2018). Subfunctionalization of the Ruby2-Ruby1 gene cluster during the domestication of citrus. Nat. Plants.

[10] Lu S.W., Ye J.L., Zhu K.J., Zhang Y., Zhang M.W., Xu Q., Deng X.X. (2021). A citrus phosphate starvation response factor CsPHL3 negatively regulates carotenoid metabolism. Plant Cell Physiol..

[11] Sun Q., He Z.C., Feng D., Wei R.R., Zhang Y., Ye J.L., Chai L.J., Xu J., Cheng Y.J., Xu Q. (2024). An abscisic acid-responsive transcriptional regulatory module CsERF110-CsERF53 orchestrates citrus fruit coloration. Plant Commun..

[12] Sun Q., He Z.C., Wei R.R., Zhang Y., Ye J.L., Chai L.J., Xie Z.Z., Guo W.W., Xu J., Cheng Y.J. (2024). The transcriptional regulatory module CsHB5-CsbZIP44 positively regulates abscisic acid-mediated carotenoid biosynthesis in citrus (*Citrus* spp.). Plant Biotechnol. J..

[13] Zhu K.J., Sun Q., Chen H.Y., Mei X.H., Lu S.W., Ye J.L., Chai L.J., Xu Q., Deng X.X. (2021). Ethylene activation of carotenoid biosynthesis by a novel transcription factor CsERF061. J. Exp. Bot..

[14] Sun Q., He Z.C., Wei R.R., Yin Y.Z., Ye J.L., Chai L.J., Xie Z.Z., Guo W.W., Xu J., Cheng Y.J. (2023). Transcription factor CsTT8 promotes fruit coloration by positively regulating the methylerythritol 4-phosphate pathway and carotenoid biosynthesis pathway in citrus (*Citrus* spp.). Hortic. Res..

[15] Zhu K.J., Chen H.Y., Mei X.H., Lu S.W., Xie H.P., Liu J.W., Chai L.J., Xu Q., Wurtzel E.T., Ye J.L. (2023). Transcription factor CsMADS3 coordinately regulates chlorophyll and carotenoid pools in *Citrus hesperidium*. Plant Physiol..

[16] Butelli E., Licciardello C., Zhang Y., Liu J., Mackay S., Bailey P., Reforgiato-Recupero G., Martin C. (2012). Retrotransposons control fruit-specific, cold-dependent accumulation of anthocyanins in blood oranges. Plant Cell.

[17] Carvalho C.M.B., Lupski J.R. (2016). Mechanisms underlying structural variant formation in genomic disorders. Nat. Rev. Genet..

[18] Alseekh S., Scossa F., Fernie A.R. (2020). Mobile transposable elements shape plant genome diversity. Trends Plant Sci..

[19] Huang Y., He J.X., Xu Y.T., Zheng W.K., Wang S.H., Chen P., Zeng B., Yang S.Z., Jiang X.L., Liu Z.S. (2023). Pangenome analysis provides insight into the evolution of the orange subfamily and a key gene for citric acid accumulation in citrus fruits. Nat. Genet..

[20] Zhou H., Ma R.J., Gao L., Zhang J.N., Zhang A.D., Zhang X.J., Ren F., Zhang W.H., Liao L., Yang Q.R. (2021). A 1.7-Mb chromosomal inversion downstream of a PpOFP1 gene is responsible for flat fruit shape in peach. Plant Biotechnol. J..

[21] Wang L., Huang Y., Liu Z.A., He J.X., Jiang X.L., He F., Lu Z.H., Yang S.Z., Chen P., Yu H.W. (2021). Somatic variations led to the selection of acidic and acidless orange cultivars. Nat. Plants.

[22] Terol J., Ibañez V., Carbonell J., Alonso R., Estornell L.H., Licciardello C., Gut I.G., Dopazo J., Talon M. (2015). Involvement of a citrus meiotic recombination TTC-repeat motif in the formation of gross deletions generated by ionizing radiation and MULE activation. BMC Genom..

[23] Wang Y.Y., Xiao Y.Q., Sun Y.T., Zhang X., Du B.Y., Turupu M., Yao Q.S., Gai S.L., Tong S., Huang J. (2023). Two B-box proteins, PavBBX6/9, positively regulate light-induced anthocyanin accumulation in sweet cherry. Plant Physiol..

[24] Wang J.H., Liu J.J., Chen K.L., Li H.W., He J., Guan B., He L. (2017). Comparative transcriptome and proteome profiling of two *Citrus sinensis* cultivars during fruit development and ripening. BMC Genom..

[25] Ren Y.J., Yang J.W., Lu B.G., Jiang Y.P., Chen H.Y., Hong Y.W., Wu B.H., Miao Y. (2017). Structure of Pigment Metabolic Pathways and Their Contributions to White Tepal Color Formation of Chinese *Narcissus* tazetta var. chinensis cv Jinzhanyintai. Int. J. Mol. Sci..

[26] Chen X.X., Gao J.H., Shen Y.Y. (2024). Abscisic acid controls sugar accumulation essential to strawberry fruit ripening via the FaRIPK1-FaTCP7-FaSTP13/FaSPT module. Plant J..

[27] Barbez E., Kubeš M., Rolčík J., Béziat C., Pěnčík A., Wang B.J., Rosquete M.R., Zhu J.S., Dobrev P.I., Lee Y. (2012). A novel putative auxin carrier family regulates intracellular auxin homeostasis in plants. Nature.

[28] Pan T.F., Kong L.C., Zhang X.X., Wang Y.H., Zhou J.Y., Fu Z.J., Pan H.L., She W.Q., Yu Y. (2022). Fruit quality and volatile constituents of a new very early-ripening pummelo (*Citrus maxima*) cultivar ‘Liuyuezao’. Front. Plant Sci..

[29] Luan Y.T., Wang S.J., Wang P., Ke F.Z., Zhu C.Q., Xu C.J. (2024). Accumulation of β-citraurin and other carotenoids in relation to the expression of PSY1 and CCD4b1 in peel of twelve red-peeled citrus cultivars. Sci. Hortic..

[30] Chen P., Liu J.B., Tang Q., Zhou T., Guo L.X., Xu Y.Y., Chai L.J., Xu Q., Deng Z.N., Li X.X. (2024). Genetic identification of citrus natural hybrid ‘Local Juhong’ using molecular marker and genomics. Genes.

[31] Wan S., Yang M., Ni F., Chen W., Wang Y., Chu P., Guan R. (2022). A small chromosomal inversion mediated by MITE transposons confers cleistogamy in Brassica napus. Plant Physiol..

[32] Fu S.L., Lv Z.L., Guo X., Zhang X.Q., Han F.P. (2013). Alteration of terminal heterochromatin and chromosome rearrangements in derivatives of wheat-rye hybrids. J. Genet. Genom..

[33] Alvarez-González L., Burden F., Doddamani D., Malinverni R., Leach E., Marín-García C., Marín-Gual L., Gubern A., Vara C., Paytuví-Gallart A. (2022). 3D chromatin remodelling in the germ line modulates genome evolutionary plasticity. Nat. Commun..

[34] Mao J.X., Wang Y., Wang B.T., Li J.Q., Zhang C., Zhang W.S., Li X., Li J., Zhang J.X., Li H. (2023). High-quality haplotype-resolved genome assembly of cultivated octoploid strawberry. Hortic. Res..

[35] Zhang F., Khajavi M., Connolly A.M., Towne C.F., Batish S.D., Lupski J.R. (2009). The DNA replication FoSTeS/MMBIR mechanism can generate genomic, genic and exonic complex rearrangements in humans. Nat. Genet..

[36] Salomoni P. (2013). Reprogramming and genome integrity: Role of non-homologous end joining. Cell Death Differ..

[37] Rogers S.O. (1994). Plant molecular biology manual D1. Extraction of Total Cellular DNA from Plants, Algae and Fungi.

[38] Li H., Durbin R. (2009). Fast and accurate short read alignment with Burrows-Wheeler transform. Bioinformatics.

[39] Van der Auwera G.A., Carneiro M.O., Hartl C., Poplin R., del Angel G., Levy-Moonshine A., Jordan T., Shakir K., Roazen D., Thibault J. (2013). From FastQ data to high confidence variant calls: The Genome Analysis Toolkit best practices pipeline. Curr. Protoc. Bioinform..

[40] Kim D., Langmead B., Salzberg S.L. (2015). HISAT: A fast spliced aligner with low memory requirements. Nat. Methods.

[41] Love M.I., Huber W., Anders S. (2014). Moderated estimation of fold change and dispersion for RNA-seq data with DESeq2. Genome Biol..

[42] Kanehisa M., Goto S. (2000). KEGG. Kyoto encyclopedia of genes and genomes. Nucleic Acids Res..

[43] Conesa A., Götz S., García-Gómez J.M., Terol J., Talón M., Robles M. (2005). Blast2go: A universal tool for annotation, visualization and analysis in functional genomics research. Bioinformatics.

