# Supplementary figures and images for: A 2.9 Mb Chromosomal Segment Deletion Is Responsible for Early Ripening and Deep Red Fruit in Citrus sinensis
Source: Int J Mol Sci. 2024 Dec 2;25(23):12931. doi: 10.3390/ijms252312931 (PMC11641548; doi:10.3390/ijms252312931)

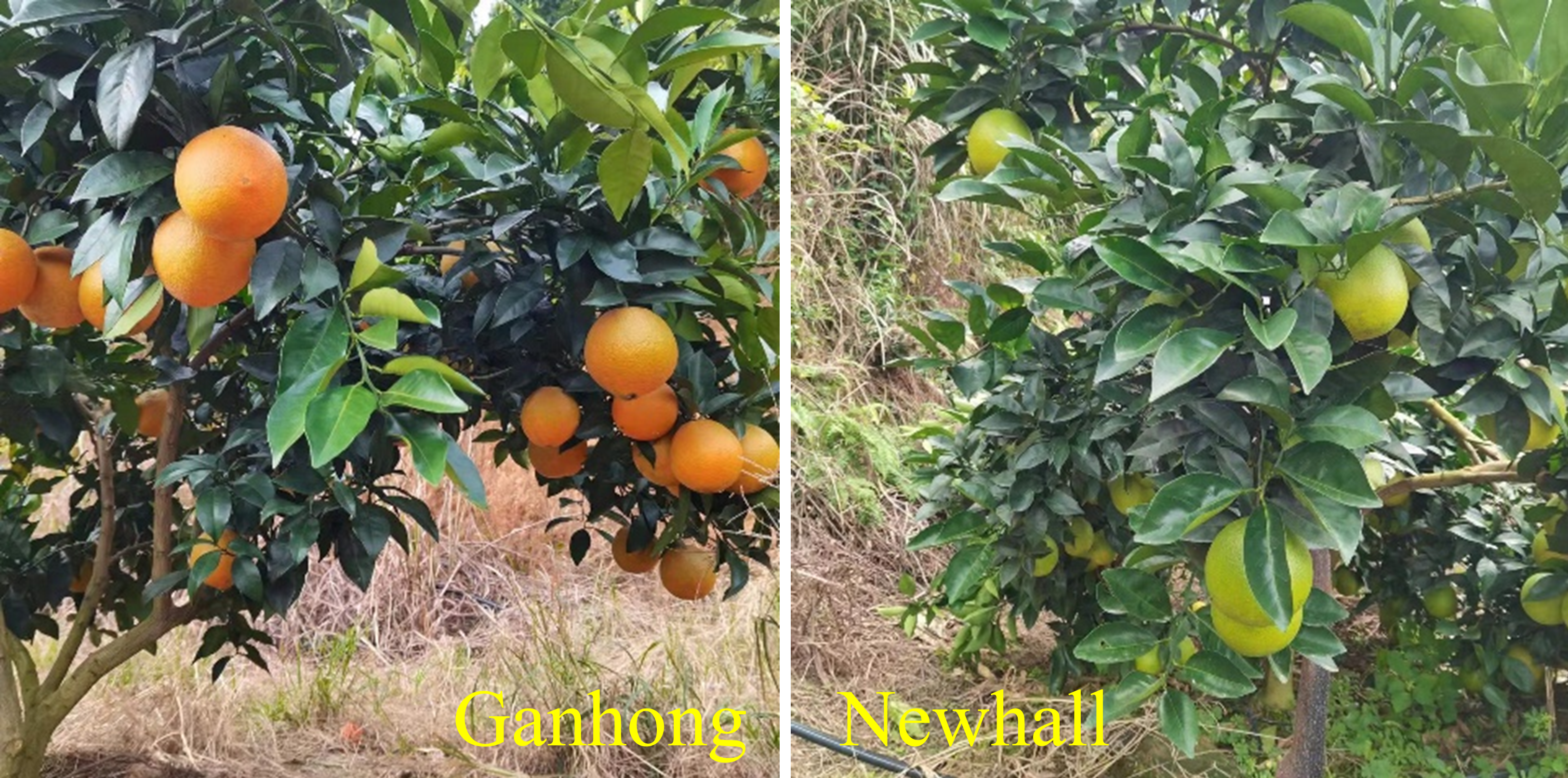

Supplement: Supplementary file 1 [file ijms-25-12931-s001.zip › Figure S1.TIF]

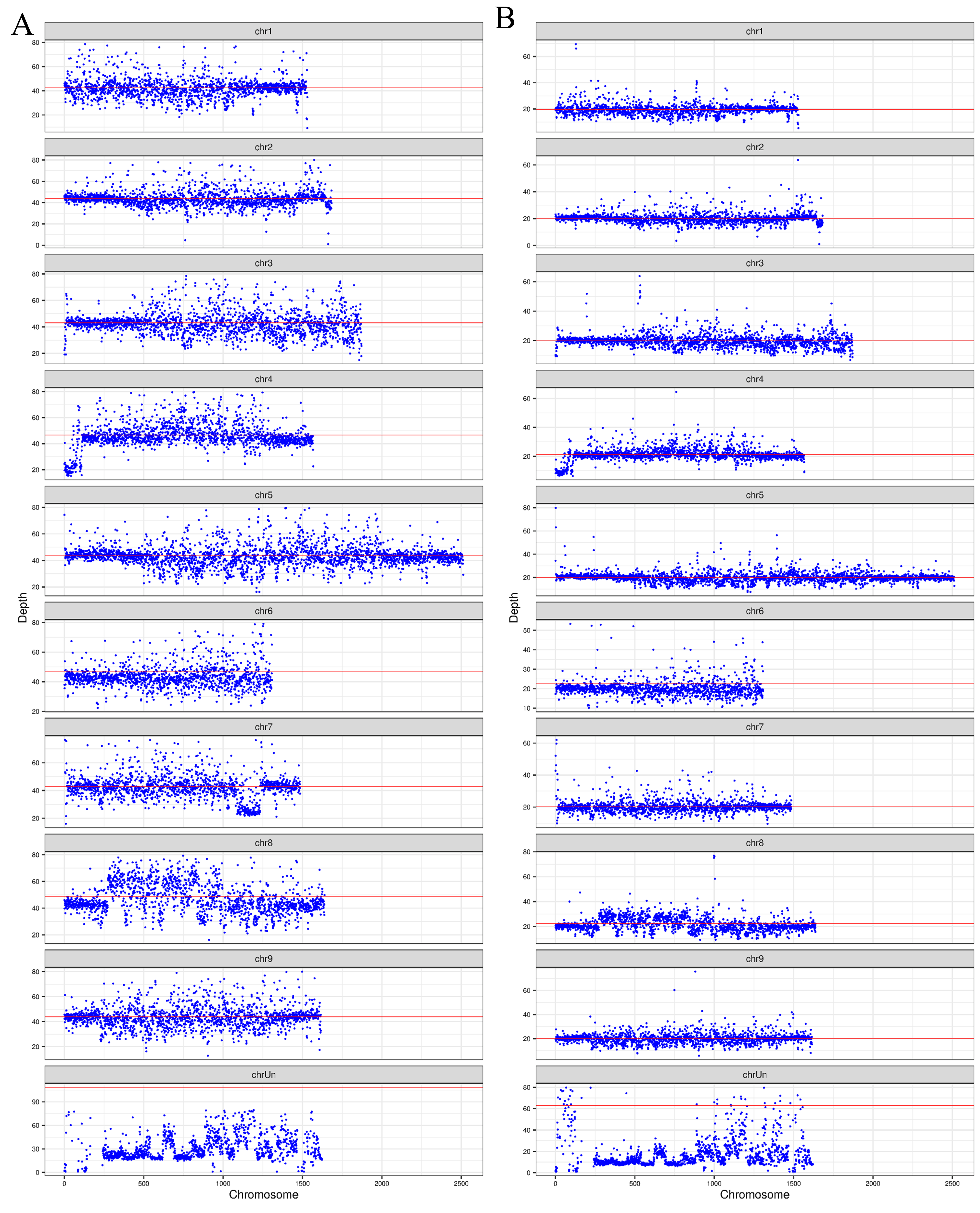

Supplement: Supplementary file 1 [file ijms-25-12931-s001.zip › Figure S2.TIF]

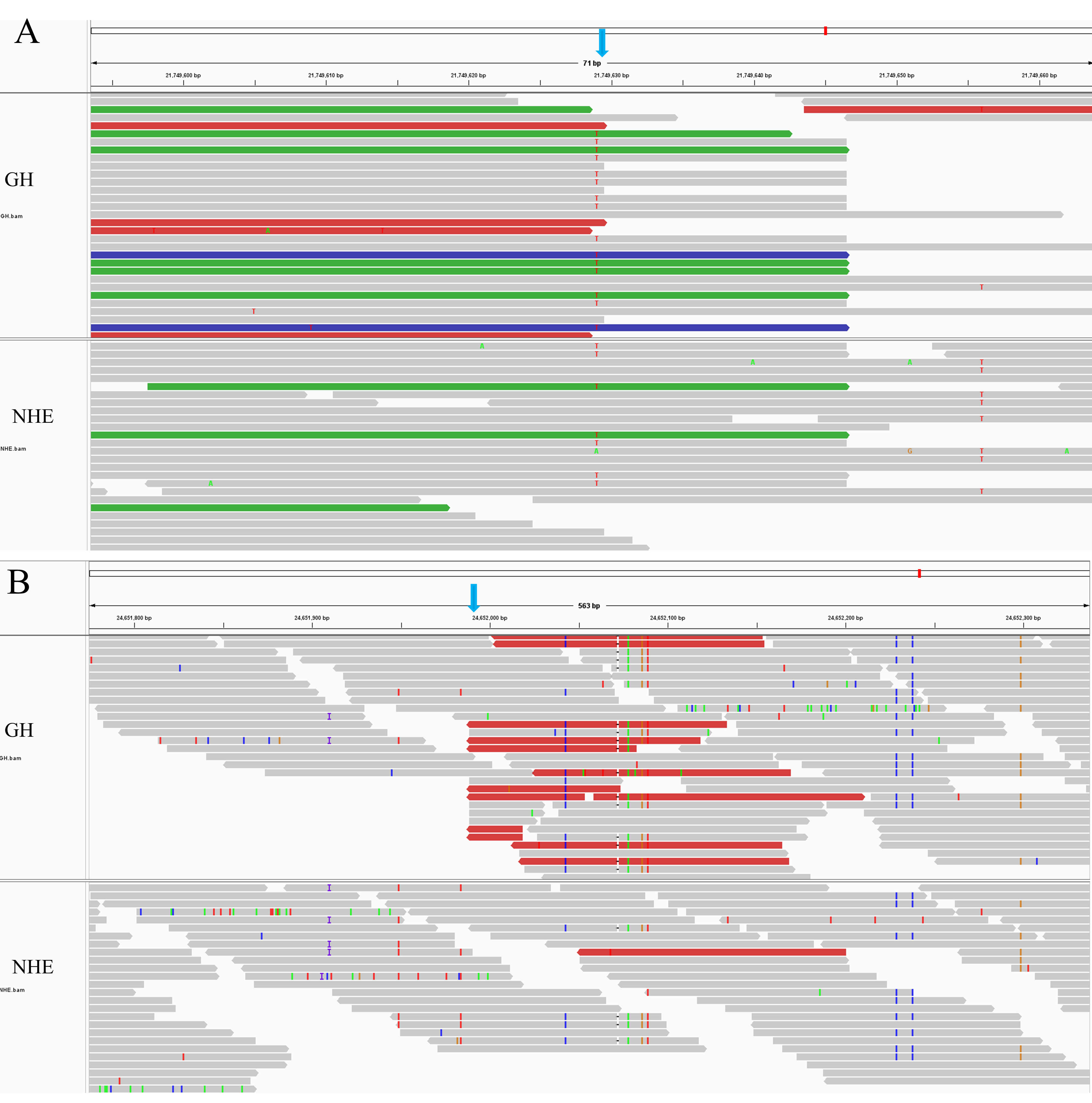

Supplement: Supplementary file 1 [file ijms-25-12931-s001.zip › Figure S3.TIF]

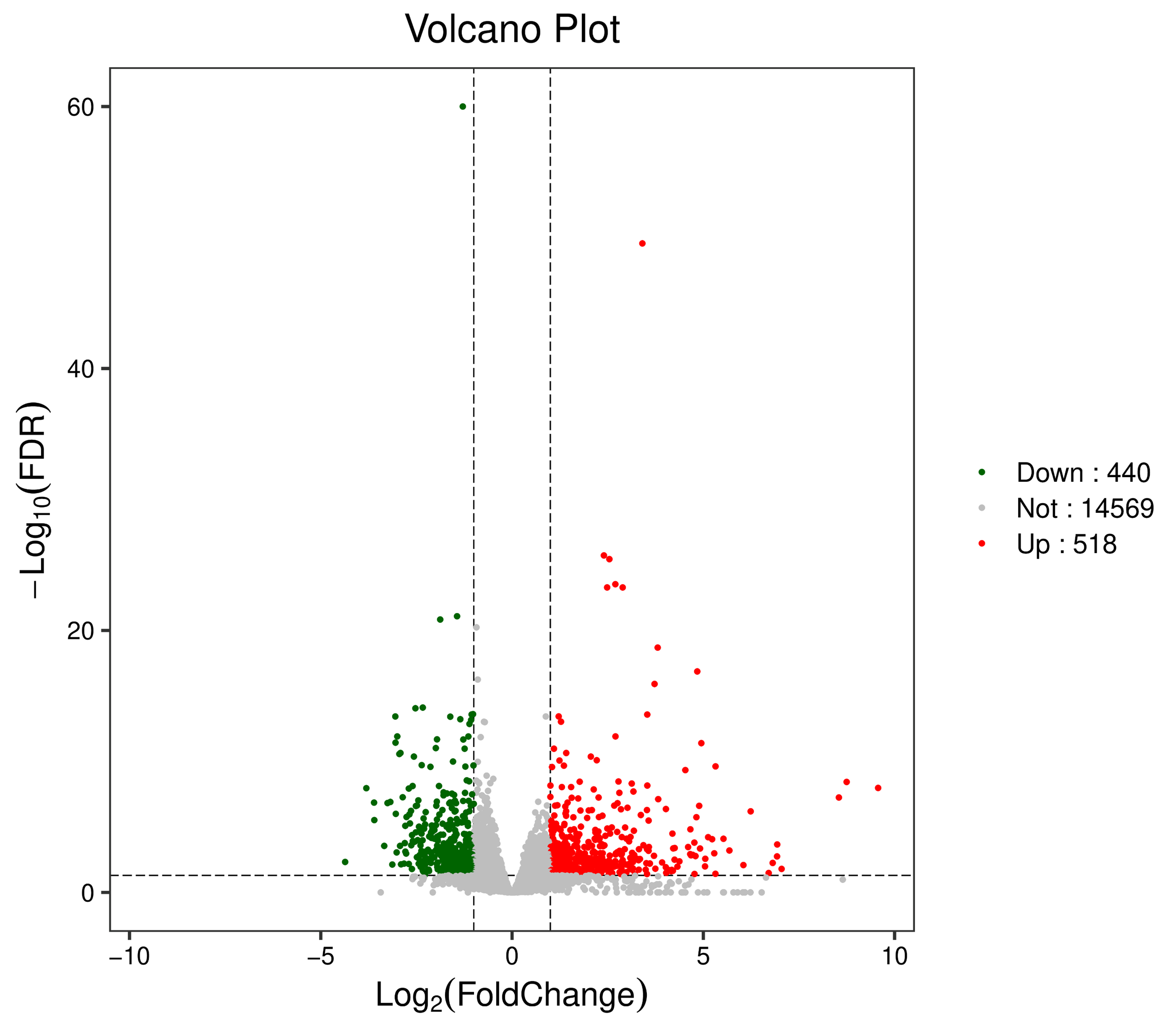

Supplement: Supplementary file 1 [file ijms-25-12931-s001.zip › Figure S4.tif]
